# Temporal and spatial multiplexed infrared single-photon counter based on high-speed avalanche photodiode

**DOI:** 10.1038/srep44600

**Published:** 2017-03-15

**Authors:** Xiuliang Chen, Chengjie Ding, Haifeng Pan, Kun Huang, Julien Laurat, Guang Wu, E Wu

**Affiliations:** 1 State Key Laboratory of Precision Spectroscopy, East China Normal University, Shanghai, 200062, China; 2 Laboratoire Kastler Brossel, UPMC-Sorbonne Universités, CNRS, ENS-PSL Research University, Collège de France, 4 Place Jussieu, 75005 Paris, France; 3 Collaborative Innovation Center of Extreme Optics, Shanxi University, Taiyuan, Shanxi 030006, China

## Abstract

We report on a high-speed temporal and spatial multiplexed single-photon counter with photon-number-resolving capability up to four photons. The infrared detector combines a fiber loop to split, delay and recombine optical pulses and a 200 MHz dual-channel single-photon detector based on InGaAs/InP avalanche photodiode. To fully characterize the photon-number-resolving capability, we perform quantum detector tomography and then reconstruct its positive-operator-valued measure and the associated Wigner functions. The result shows that, despite of the afterpulsing noise and limited system detection efficiency, this temporal and spatial multiplexed single-photon counter can already find applications for large repetition rate quantum information schemes.

Photon-number-resolving (PNR) detectors play a critical role in various applications ranging from experiments associated to the foundations of quantum mechanics to quantum information technologies^1^,^2^,^3^. For instance, PNR detectors provide a powerful tool for quantum state engineering and quantum light source characterization^4^. In the linear optical quantum computation scheme^5^ proposed by Knill, Laflamme, and Milburn, PNR detectors are also ultimately required to resolve the photon number of the involved optical resources. The PNR ability would as well improve security of some quantum key distribution schemes against photon-number-splitting attacks^6^.

Up to date different types of PNR detectors have been developed, including the superconducting transition-edge-sensor (TES)^7^ with full PNR capability, and detectors with some PNR capability^8^, such as photo-multiplier tubes^9^, quantum-dot field-effect transistors^10^, multipixel counters^11^,^12^,^13^ and superconducting nanowires^14^,^15^. In parallel, a large effort has also been dedicated to realize PNR capability based on single-photon avalanche photodiodes (APDs) due to their advantages, such as low cost and easy operation at room temperature^2^,^16^,^17^,^18^,^19^,^20^,^21^,^22^,^23^,^24^. Normally APDs can only discriminate between zero and at least one photon, i.e. working as “on/off” detector. To resolve higher photon numbers, two main techniques have been used. One is based on a sub-saturation operation of APDs, which enables to output a detectable signal proportional to the incident photon number with the help of spike noise cancellation method^16^,^21^. However, this operation needs critical optimization of the detector parameters^17^. The other method relies on the simultaneous use of multiple APDs by temporal or spatial multiplexing^18^,^19^,^20^,^24^,^25^,^26^,^27^. This approach provides a simple, effective and low-cost way to achieve a PNR capability. In this context, increasing the detection rate is an important challenge.

In this paper, we demonstrate a high-speed temporal and spatial multiplexed infrared single-photon counter using a dual-channel 200 MHz single-photon detector based on InGaAs/InP APDs. Temporal and spatial multiplexing is realized with a fiber loop and two 50/50 beam-splitters. With a single loop, detection up to 4 photons is possible, and the PNR capability can also be improved just by adding more cascaded fiber loops, instead of adding more detectors. In comparison to the multiplexed InGaAs/InP APD detector reported in ref. 20 for which the repetition rate of the light source was kept at 90 kHz due to the deadtime of the detector, our scheme takes benefit from the self-differencing technique^28^,^29^, and enables to reach a gating repetition rate up to 200 MHz with a deadtime as short as 20 ns. Meanwhile the dark count noise is limited to 8 × 10^−6^ per gate and the afterpulsing probability is kept around 2.5% for both channels when the detection efficiency is around 10%. As there was few effective way to estimate the influence of the afterpulsing noise in the PNR detection, quantum tomography^25^,^26^,^30^,^31^,^32^,^33^,^34^,^35^,^36^ of the realized single-photon counter is presented and we provide the reconstructed positive operator-valued measure (POVM)^37^, which fully characterizes the PNR capability of the detector. The Wigner function for each possible outcome is calculated from the reconstructed POVM elements.

## Results and Discussion

The temporal and spatial multiplexed single-photon counter can be fully characterized by its POVM^30^, which is a set of operators 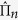 corresponding to a particular measurement outcome n. Since the temporal and spatial multiplexed single-photon counter considered here is phase-insensitive, its POVM is thus diagonal in the Fock state basis and given by


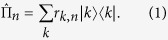


The coefficient 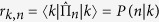 is the conditional probability to obtain n clicks given an impinging Fock state |*k*〉. All the coefficients *r*_*k,n*_ can be determined without any a priori assumptions about the detector subject to calibration by using the so-called quantum detector tomography (QDT). Specifically, we can probe the response of the device with a set of coherent states |*α*_*j*_〉. In principle, the probabilities for each outcome can be calculated by


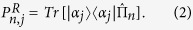


On the other hand, these probabilities 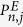 can also be measured experimentally. Therefore, the POVM elements can be estimated by the optimization problem 
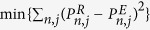
. The reconstruction process can be implemented by a maximum-likelihood algorithm, which guarantees that the reconstructed POVM meets the required positivity constraints^38^. There is no regularization function used in the maximum-likelihood algorithm. The essential part of this algorithm is to construct the so-called iteration operator for each iteration step, in which the positivity of POVM element is preserved.

**Figure 1:**
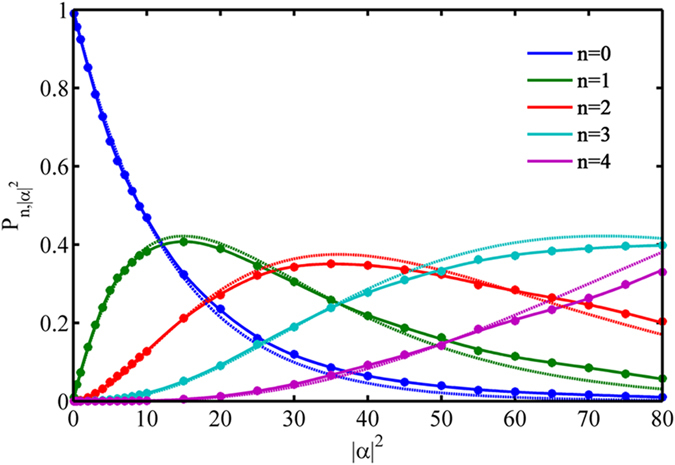
Experimental data. The plots provide the probability of the different detector outcomes as a function of the average photon number |*α*|^2^ of the weak coherent probe pulses. The points correspond to the experimental data while the solid and dotted lines provide the results of the tomography and the theoretical simulation respectively. (blue for *n* = 0, green for *n* = 1, red for *n* = 2, cyan for *n* = 3, purple for *n* = 4).

Figure 1 shows the raw experimental data of the QDT. It provides the probability of each possible detection outcomes as a function of the average mean photon number in the probe pulse. From these measurements, the POVM elements are reconstructed. The fidelity^20^ of the probability distributions obtained from the reconstructed POVM with the measured ones for different average photon numbers is calculated by


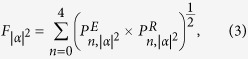


where 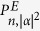 and 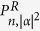 are the probability of detected photon number state n from the experimental data and reconstructed POVM, while the incident average photon number is |*α*|^2^. For all the |*α*|^2^ from 0 to 80, the values of 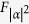 are all above 99.99%, indicating a very good agreement between the experimental tomography data and the reconstructed POVM.

As mentioned in ref. 24, the full characterization of afterpulsing was complicated^22^,^23^ and by prolonging the deadtime and shortening the gating pulse duration applied on the APD the afterpulsing noise effect could finally be neglected. However, as an unavoidable noise source, in some applications, the afterpulsing noise could not be ignored, especially when the incident photon intensity is high. A theoretical model has been developed only taking into account the detection efficiency and the dark count probability^25^,^26^,^30^,^31^. According to the comparison between the simulation and the reconstructed data shown in Fig. 1, it can be noticed that when the incident photon intensity is below 20, the two curves match better than the ones with higher incident photon number. The deviation is mainly due to the fact that the model does not consider the afterpulsing noise, which is dependent on the number of the photon-induced avalanche. However, the QDT can characterize the detector with a black box approach. By preparing a collections of known states, which are coherent states in our scheme, and recording the outputs of the detector, we could get the full characterization of the detector described with POVM elements, without requiring any other information of the detector such as detection efficiency, dark counts and afterpulsing noise. Therefore, QDT could characterize the detector more accurately when unexpected parameters are in play.

**Figure 2:**
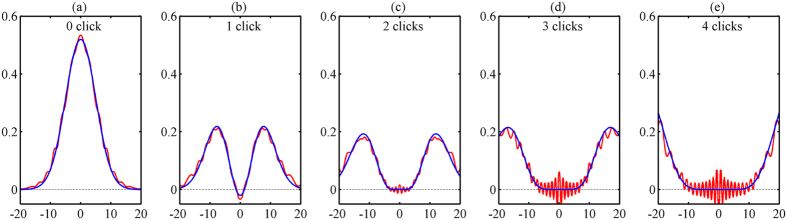
(**a**–**e**) Cross section of the experimental Wigner functions obtained from the reconstructed POVM elements for 0 to 4 clicks outcomes. The red and blue lines provide the experimental Wigner function and the theoretical simulation respectively. Note that all the Wigner functions are normalized between −1 and 1.

The reconstructed POVM elements can also be represented by their associated Wigner functions, which is a quasi-probability distribution. In contrast to classical probability distributions, they can take negative values, which are considered as a strong signature of the full-quantum character of the measurement device under study. The Wigner function corresponding to the POVM operator 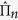 is given by


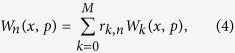


where M corresponds to the truncation photon-number for the reconstruction process and *W*_*k*_(*x, p*) is the Wigner function corresponding to the Fock state |*k*〉 given by


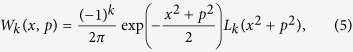


where *L*_*k*_ is the Laguerre polynomial. Due to the fact that the detector is phase-insensitive, the Wigner functions are rotationally symmetric with respect to their center. In Fig. 2(a–e) the cross-section for each Wigner function associated to the reconstructed POVM elements is given to illustrate the agreement between the reconstructed POVMs and the theoretical simulations. Specifically, for one-click POVM the negativity at the origin of the Wigner function is −0.026 (ideally −1), indicating the quantum feature of the detector. For more click POVMs, the bottom of the Wigner functions is flattened with a very small negativity, which is due to the fast decoherence in a lossy and noisy environment^25^. It is worth noting that ripples appearing in the Wigner functions for 3- and 4-click POVMs are just an edge effect due to number state truncation^26^.

## Conclusion

In conclusion, we have demonstrated a high-speed temporal and spatial multiplexed infrared single-photon counter by combining a fiber-assisted loop and a 200 MHz dual-channel detector based on InGaAs/InP APD with self-differencing technique. This temporal and spatial multiplexed single-photon counter could detect up to 4 photons. In order to characterize the temporal and spatial multiplexed single-photon counter, the POVM and associated Wigner functions are calculated and compared to the theoretical model, showing a good agreement. By increasing the quantum efficiency of the InGaAs/InP APD and reducing the overall losses of the fiber splitting scheme, better resolution capability could be achieved. This capability can also be improved by adding more cascaded fiber loops.

## Methods

**Figure 3:**
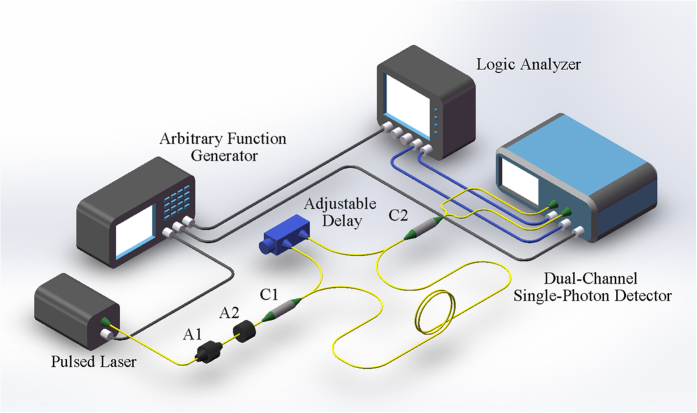
Experimental setup. The pulsed laser at 1550 nm is operated with a repetition rate of 10 MHz. It is strongly attenuated to be used as QDT probe. The delay between the two paths in the fiber loop is adjusted to be 50 ns. The dual-channel single-photon detector based on InGaAs/InP APD is operated at 200 MHz, and the self-differencing technique is used to suppress the spike noise induced by the gating pulse. The outputs of the detector are recorded by the logic analyzer. The synchronization of the whole system is provided by the arbitrary function generator. A1: adjustable attenuator; A2: fixed attenuator; C1 and C2: 50/50 fiber beam-splitters.

The experimental setup is sketched on Fig. 3. The temporal and spatial multiplexed single-photon counter is composed of a dual-channel single-photon detector and a fiber loop consisting of two 50/50 fiber couplers. An adjustable delay inserted into the short path enables to precisely control the delay between the two. A pulsed 1550 nm laser with a 10 MHz repetition rate and 19 ps pulse duration is used for probing the detector. The laser pulses are sent to a fixed calibrated attenuator and an adjustable attenuator that is used to adjust the average photon number of the weak incident optical pulse. The average photon number per pulse can be varied from 0 to 80. The outputs of the second 50/50 fiber beam-splitter are detected. A logic analyzer (Tektronix TLA6401) is connected to the output of the dual-channel detector to acquire the timing information of the detected photons. The pulsed laser, the dual-channel single-photon detector and the logic analyzer are synchronized with a 10-MHz signal from an arbitrary function generator (Tektronix AFG3102).

**Figure 4:**
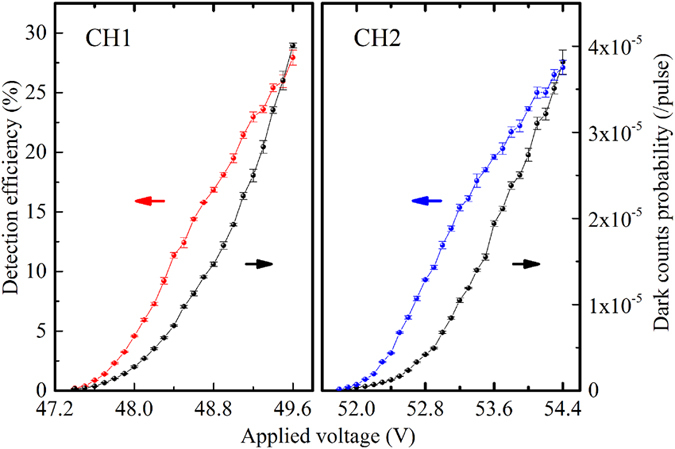
Detection efficiency and dark count probability for each channel of the dual-channel high-speed detector as a function of the voltage applied on the APD.

The dual-channel detector relies on two InGaAs/InP APDs operated in gated Geiger mode at −40 °C to reduce the dark count noise. With the self-differencing technique, the spike noise generated by the charging and discharging process on the APD is suppressed. By subtracting the identical signal in the APD response of the two successive gating cycles, the weak avalanche signal can be extracted from the spike noise. The spike noise suppression ratio of each channel of the single-photon detector is up to 25 dB. The operation repetition rate of the detector is fixed at 200 MHz and synchronized with the 10 MHz signal of the arbitrary function generator with an internal phase locked loop. The gating pulse duration is about 900 ps and the deadtime of each channel is set to be about 20 ns. Figure 4 shows the detection efficiency and the dark count of each channel for different voltages applied on the APDs. As it can be seen, the maximum detection efficiency of the two channels CH1 and CH2 are about 29% and 27%, respectively. However, the dark count noise increases rapidly to ~10^−5^ per gate when the detection efficiency is high. To limit the effect of dark noise on the PNR ability, we compromise the detection efficiency for a lower dark count noise. The dark count probability of CH1 decreases to 8.5 × 10^−6^ per gate at detection efficiency of 11%, while CH2 is 7.4 × 10^−6^ per gate at 12% at the gating repetition rate of 200 MHz. The afterpulsing probability is measured to be about 2.5% for both channels.

Crosstalk between the two channels is another kind of noise causing error counts in the detection. To evaluate the crosstalk, the photon source is connected to one of the channels, while the other is kept open. When the incident photon number increases from 1 to 50 photons/pulse on one channel, the open channel exhibits the same dark count rate, indicating that the two channels work independently and that the crosstalk between the two is negligible.

When passing through the fiber loop, an optical pulse is split into four time-bins at the output. The temporal and spatial multiplexed detector has therefore five possible outcomes: no click, one click, two clicks, three clicks and four clicks. As the dual-channel detector is operated in the gated Geiger mode and the deadtime is set at 20 ns, the delay between the two paths is precisely controlled to be 50 ns in order to ensure all the pulses can be detected. The logic analyzer sorts the outputs to 5 events according to the arrival channel and timing information to produce the 0 to 4 outcomes. The overall detection efficiency of temporal and spatial multiplexed single-photon counter, including the losses of the fiber loop, is about 7.7%.

The theoretical model has been developed taking into account the detection efficiency and the dark count probability with an assumption of identical detectors and prefect 50:50 beam-splitters^25^,^26^,^30^,^31^.The coefficients *r*_*k,n*_ can be expressed as


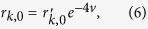



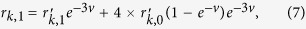



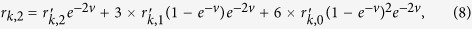



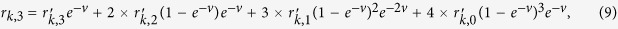



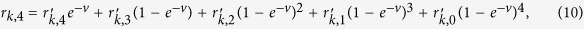


where *ν* is the dark count probability per pulse, and 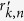 are the coefficients of the theoretical model in the absence of dark count, which can be written as


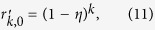



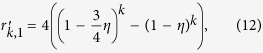



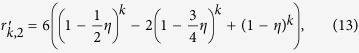



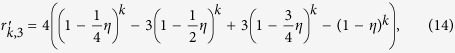



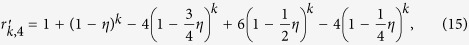


where *η* is the detection efficiency of the detector.

## Additional Information

**How to cite this article:** Chen, X. *et al*. Temporal and spatial multiplexed infrared single-photon counter based on high-speed avalanche photodiode. *Sci. Rep.* **7**, 44600; doi: 10.1038/srep44600 (2017).

**Publisher's note:** Springer Nature remains neutral with regard to jurisdictional claims in published maps and institutional affiliations.
